# Development of a pHrodo-Based Assay for the Assessment of *In Vitro* and *In Vivo* Erythrophagocytosis during Experimental Trypanosomosis

**DOI:** 10.1371/journal.pntd.0003561

**Published:** 2015-03-05

**Authors:** Benoit Stijlemans, Jennifer Cnops, Peter Naniima, Axel Vaast, Viki Bockstal, Patrick De Baetselier, Stefan Magez

**Affiliations:** 1 Laboratory of Cellular and Molecular Immunology, Vrije Universiteit Brussel, Brussels, Belgium; 2 Department of Myeloid Cell Immunology, Vlaams Instituut voor Biotechnologie (VIB), Brussels, Belgium; 3 Department of Structural Biology, Vlaams Instituut voor Biotechnologie (VIB), Brussels, Belgium; New York University School of Medicine, UNITED STATES

## Abstract

Extracellular trypanosomes can cause a wide range of diseases and pathological complications in a broad range of mammalian hosts. One common feature of trypanosomosis is the occurrence of anemia, caused by an imbalance between erythropoiesis and red blood cell clearance of aging erythrocytes. In murine models for *T*. *brucei* trypanosomosis, anemia is marked by a very sudden non-hemolytic loss of RBCs during the first-peak parasitemia control, followed by a short recovery phase and the subsequent gradual occurrence of an ever-increasing level of anemia. Using a newly developed quantitative pHrodo based *in vitro* erythrophagocytosis assay, combined with FACS-based *ex vivo* and *in vivo* results, we show that activated liver monocytic cells and neutrophils as well as activated splenic macrophages are the main cells involved in the occurrence of the early-stage acute anemia. In addition, we show that trypanosomosis itself leads to a rapid alteration of RBC membrane stability, priming the cells for accelerated phagocytosis.

## Author Summary

Extracellular trypanosomes, causative agents of sleeping sickness and Nagana, threaten human and animal health throughout the world. Anemia is a hallmark feature of virtually every type of trypanosome infection. During the early phase of experimental murine trypanosomosis, acute anemia occurs as witnessed by a 50% reduction in red blood cells within a 48 hour time span. The acute nature of this phenomenon suggests the implication of a consumptive process such as erythrophagocytosis. However, due to the multiple significant drawbacks of the presently used phagocytosis techniques, this has never been straightforwardly demonstrated. Here we developed a new erythrophagocytosis assay based on the labeling of red blood cells with the acid-sensitive dye pHrodo. This assay unequivocally distinguishes erythrophagocytozing cells *in vivo* and *in vitro* via flow cytometry and fluorescent microscopy. Using this new assay, we show that the acute anemia during experimental trypanosomosis is a result of enhanced erythrophagocytosis by activated liver monocytic cells and neutrophils as well as by activated splenic macrophages. Moreover, the red blood cell membrane composition and stability are altered during the infection, priming them for enhanced clearance by the myeloid phagocytic system.

## Introduction

Extracellular trypanosomes including *Trypanosoma brucei*, *T*. *evansi*, *T*. *congolense* and *T*. *vivax*, are parasites that affect a very broad host range, and combined, threaten human and animal health throughout various continents. Despite the incredibly wide range of trypanosomosis-associated diseases and pathological complications, one common feature of trypanosomosis is the occurrence of anemia, which is seen in human infections [1] as well as non-human primate trypanosomosis [2,3], and most other wildlife and livestock trypanosome infections [4]. Anemia is a condition in which an imbalance occurs between erythropoiesis and red blood cell clearance. RBC are either destined for clearance (e.g. by senescence, antibody coating or damage) or are cleared as “innocent bystanders” (e.g. during hemorrhage) [5]. A loss of membrane phospholipid asymmetry has been recognized as a key trigger that can lead to recognition and extravascular removal of senescent RBCs, disordered RBCs, and transfused RBC that have been stored for a long time, by cells of the myeloid phagocyte system [6–8]. Hence, infection-associated complications that affect either of these processes will lead to anemia. Trypanosomosis is suggested to 1) hamper erythropoiesis, 2) enhance erythrophagocytosis (also termed extravascular hemolysis) and 3) in some cases, eg *T*. *vivax* infections, cause intravascular hemolysis. In murine models for *T*. *brucei* trypanosomosis, anemia is marked by a very sudden non-hemolytic loss of RBCs during the first-peak parasitemia control, followed by a short recovery phase and the subsequent gradual occurrence of an ever increasing level of anemia, reminiscent of ‘anemia of chronic infection’ [9–13]. Interestingly, as anemia occurs in B-cell deficient μMT mice with similar kinetics as WT mice, the process involved appears antibody independent [14,15]. This contrasts a previous *in-vitro* based hypothesis that cross-reactive anti-VSG antibodies might contribute to a complement-mediated hemolysis event [16]. Based on combined recent data, the most plausible explanation for the initiation of trypanosomosis-associated anemia is the occurrence of enhanced RBC phagocytosis, resulting from a pro-inflammatory cytokine storm occurring during the early stage of infection, leading to macrophage hyper-activation and enhanced erythrophagocytosis [12,13,17–20]. However, till now two main obstacles have hampered the in depth assessment of this hypothesis as (i) previous methods for RBC phagocytosis have difficulties differentiating between actual RBC uptake and RBC adherence to phagocytozing cells, and (ii) quantification of phagocytozed RBC numbers with simultaneous characterization of *in vivo* RBC phagocytozing cells has been virtually impossible. In order to address these issues, we now used a newly developed pHrodo based *in vitro* erythrophagocytosis assay, as well as an *ex vivo* FACS based analysis using the same substrate. Unique in this approach is that the visualization of RBC labeling is pH dependent and only becomes traceable in the acidic environment of the lysosome of phagocytozing cells. Hence, we were able to show *ex vivo* that activated liver monocytes, monocyte-derived macrophages as well as neutrophils are the main cells contributing to trypanosomosis-associated acute stage erythrophagocytosis. In addition, we show that trypanosomosis itself leads to a rapid alteration of RBC membrane stability, priming the cells for accelerated phagocytosis.

## Materials and Methods

### Mice

7–8 week old female C57Bl/6 mice purchased from Janvier as well as ubiquitin-GFP (Jackson Laboratories) mice bred in-house were housed at the animal facility of the Vrije Universiteit Brussel.

### Ethics statement

All experiments complied with the ECPVA guidelines and were approved by the ETHICAL COMMITTEE for ANIMAL EXPERIMENTS (ECAE) at the Vrije Universiteit Brussel (protocol #14–220–23 and #12–220–2).

### Parasites and anemia development

Mice were infected by intraperitoneal (i.p.) injection of 5000 pleomorphic *Trypanosoma brucei* AnTat1.1E parasites, which were a kind gift from N. Van Meirvenne (Institute for Tropical Medicine, Belgium). RBC counts were determined via a hematocytometer at two-day intervals on 2,5μl blood sample collected from the tail vein of non-infected and infected animals. Anemia was expressed as the percentage of reduction in RBC counts compared to non-infected animals.

### Cell isolation and culturing (erythrophagocytosis assay)

**Fig 1:**
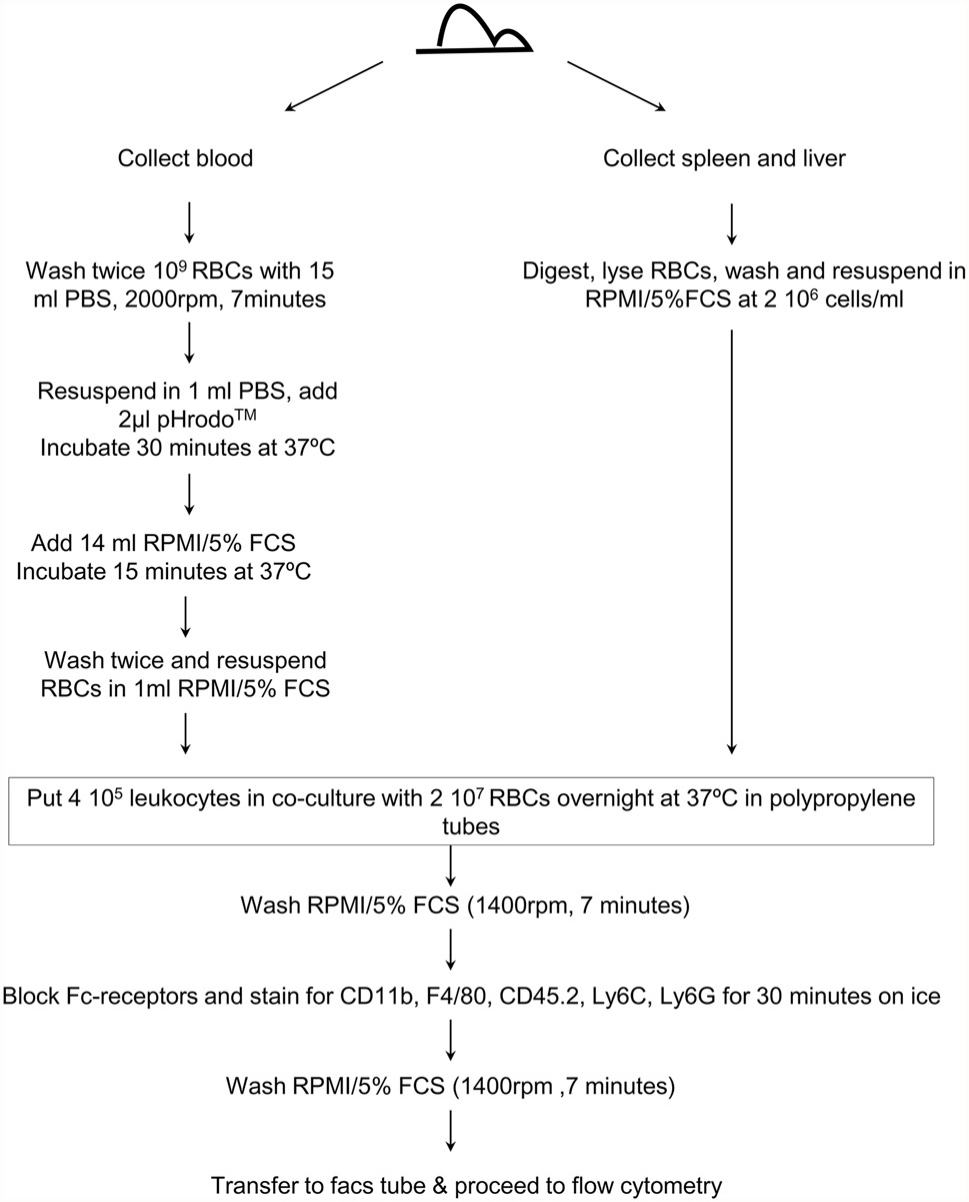
Flow chart of the *in vitro* erythrophagocytosis assay protocol.

Peritoneal exudate cells (PECs), spleen and liver were harvested from CO_2_ euthanized non-infected and day 6 infected mice. Livers were minced in 10 ml digestive media (0.05% collagenase type A in Hanks’ Balanced Salt Solution (HBSS) without calcium or magnesium; Invitrogen) and incubation at 37°C for 30 minutes, the digested tissue was homogenized and filtered (40 μm pore filter). Spleen cells were obtained by homogenizing the organs in 10 ml RPMI medium containing 5% foetal calf serum (FCS) with or without collagenase type A, Next, the liver and spleen cell suspension was centrifuged (7 minutes, 300×*g*, 4°C) and the pellet treated with RBC lysis buffer (0.15 M NH_4_Cl, 1.0 mM KHCO_3_, 0.1 mM Na_2_-EDTA). Subsequently, the cells were resuspended in ME—medium (RPMI medium, 5% FCS, 1% L-glutamine and non essential amino acids, 1% Penicillin-Streptomycin and β-mercaptoethanol) and 4 10^5^ cells were put in co-culture with or without 2 10^7^ labeled or unlabeled RBCs in polypropylene tubes (BD Biosciences). Co-cultures were incubated overnight at 37°C and 5% CO_2_ with or without lipopolysaccharide (LPS) stimulation (1μg/ml). An overview of the isolation protocol and erythrophagocytosis assay is given in Fig. 1.

### *In vivo* erythrophagocytosis assay setup

7–8 week old female C57Bl/6 non-infected or *T*. *brucei* infected (day 6 p.i.) mice were injected intravenously (i.v.) with 10^9^ pHrodo labeled or unlabeled RBCs (from either C57Bl/6 or Ubiquitin-GFP mice) in 200 μl PBS. After 18 hours, mice were CO_2_ euthanized and spleen and livers were isolated and processed into single cell suspension as described above. Next, the cells were analyzed via flow cytometry as described further.

### Red blood cell labeling

Blood was harvested from CO_2_ euthanized mice by cardiac puncture using 50μl 1000 U/ml heparin. RBCs were counted and 10^9^ RBCs were washed twice with 15 ml PBS, 2000 rpm, 7 minutes. Next, RBCs were labeled with 2 μl pHrodo Red succinimidyl ester (pHrodo Red, Life Technologies) in a final volume of 1 ml PBS for 60 minutes at 37°C. Subsequently, labeled RBCs were incubated for 15 minutes with 10 ml RPMI/5% FCS at 37°C and washed twice with the same medium, 2000 rpm, 7 minutes. Labeled RBCs were resuspended in RPMI/5% FCS at a final concentration of 10^9^ RBCs/ml and put in co-culture with isolated leukocytes (20μl blood/condition). As negative control, RBCs were treated in the same manner without addition of the pHrodo dye.

### Flow cytometric analysis

After overnight co-culturing (see above) cells were subjected to flow-cytometrical analysis. Briefly, the cells were washed with FACS medium (5% FCS in RPMI) and non-specific binding sites were blocked by incubating 20 minutes at 4°C with an Fc-blocking antibody (anti-CD16/32, clone 2.4G2). Next, cell suspensions were stained with fluorescent conjugated antibodies for 30 minutes at 4°C. Fluorescent antibodies: CD11b PE-Cy7 clone M1/70, F4/80 FITC clone C1:A3-A, Ly6C APC clone AL-21, Ly6G PerCP-Cy5.5 clone 1A8, CD45 APC-Cy7 clone 30-F11 (BD Biosciences), CD64 PE clone X54–5/7.1. (BioLegend), CCR2 PE clone 475301, MerTK PE clone 108928 (R&D systems), Ly6B clone 7/4 (AbD Serotec). Following washing with FACS buffer they were analyzed on a FACS Canto II flow cytometer (BD Biosciences) and data was processed using FlowJo software (Tree Star Inc.).

### Erythrocyte osmotic fragility

The osmotic fragility of erythrocytes was determined using the adjusted protocol from Meurs et al. [21]. Solutions with decreasing concentrations of NaCl were prepared by mixing distilled H_2_O and HBSS solutions. These solutions change in isotonicity as the NaCl concentration decreases, resulting in hemolysis of a fraction of the erythrocytes and the red coloration of the solution (hemoglobin content) can be measured spectrophotometrically. A gradient of solutions was prepared and total hemolysis was determined by exposure to 100% distilled H_2_O, 0% hemolysis was determined by exposure to 100% HBSS-solution. 3μl of blood was added to 300μl of each solution, using flat bottom 96 well plates (BD Biosciences). To determine if the labeling had an effect on RBC fragility, also pHrodo labeled RBCs and unlabeled RBCs analyzed in the same manner. After mixing by gently pipetting up and down, the solutions were incubated at room temperature for 20 minutes. Subsequently, erythrocytes (remnants) were pelleted by centrifugation at 2000 rpm for 10 minutes and 150μl supernatant was transferred to a new 96 well plate. The absorbance of the solutions was determined at 550 nm. The percentage of hemolysis was plotted against the concentration of NaCl in the medium and the NaCl concentrations corresponding with 50% hemolysis were determined. OD’s were normalized by using the smallest value of the data sets as 0% (this is the OD measured in PBS) and 100% is defined as the largest value of the data sets (OD measured in H_2_O).

### Fatty acid analysis of the erythrocyte membrane

The fatty acid composition of RBCs was analyzed by gas chromatography. RBCs from non-infected and *T*. *brucei* infected mice were stained for Ter119 PE (clone TER-119) and CD71 APC (clone R17217) (BD biosciences). Next, 10^7^ Ter119^+^ CD71^-^ RBCs were sorted (FACS Aria^TM^) and lyophilized (Flexi-dry μP Microprocessor control, FTS^TM^ systems) according to the manufacturers protocol. Subsequently, fatty acids were extracted and methylated using a method described elsewhere [22] and analyzed by gas chromatography.

### Statistical analysis

Statistical analysis was performed using Student-test and GraphPad Prism software (GraphPad 6, San Diego, CA). Values are expressed as mean ± standard error mean (SEM). Values of p≤ 0.05 are considered to be statistically significant.

## Results

### Set-up of the pHrodo *in vitro* erythrophagocytosis assay

**Fig 2:**
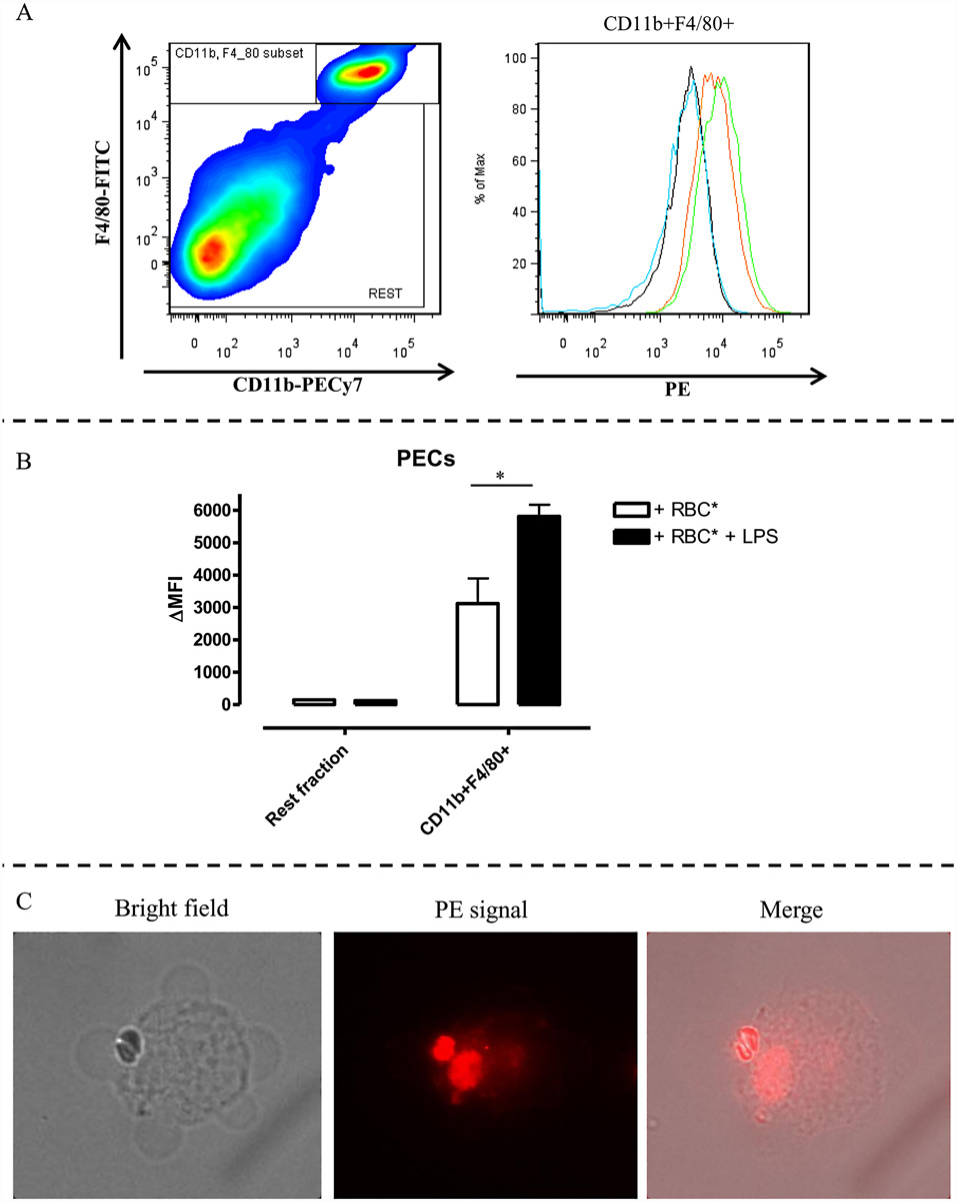
pHrodo erythrophagocytosis assay using naïve PECs. A (left panel): CD11b versus F4/80 profile following gating on CD45+ cells allows identifying two distinct populations; (i) CD11b^+^F4/80^+^ cells which represent macrophages within the PEC population and (ii) cells which are negative or low for CD11b and F4/80 expression referred by as the Rest fraction. A (right panel): histogram showing the PE signal obtained following gating on CD11b^+^ F4/80^+^ cells when PECs are incubated alone (black line), with unlabeled RBCs (blue line), with pHrodo-labeled RBCs (orange line) or stimulated with LPS and incubated with pHrodo-labeled RBCs (green line). B: Histogram showing delta medium fluorescence intensity (ΔMFI) obtained by subtracting the PE signal for cells incubated with unlabeled RBCs from cells incubated with pHrodo-labeled RBCs. PECS were unstimulated (white bars) or stimulated with 1μg/ml LPS (black bars). Results are presented +/- SEM and are representative of 4 independent experiments (for each point triplicates were used). (*: p-values ≤ 0.05) C: Microscopic pictures from PECs incubated with pHrodo-labeled RBCs.

Multiple assays for erythrophagocytosis have been developed in the past, each set-up having its own drawbacks such as the need for radioactive reagents. Here we use the pHrodo dye for the labeling of RBCs. The dye reacts with the primary amines on the RBC to yield a covalently linked pH probe, which increases in fluorescence as the pH of the surroundings becomes more acidic. Due to the low pH of the phagolysosome, phagocytozed RBCs can be visualized without being mistaken for RBCs merely sticking to the outside of the phagocytozing cell. Hence this technique enables the straightforward quantification of erythrophagocytosis using FACS and, if required, confirmation of the obtained result by fluorescence microscopy. As shown in Fig. 1, we used this technique to monitor erythrophagocytosis in different cellular contexts, always using the same experimental layout. To validate this erythrophagocytosis assay, pHrodo labeled RBCs and PECs from non-infected mice were put in co-culture overnight with or without LPS stimulation and subsequently stained and analyzed via flow cytometry to distinguish phagocytozing cells. Following gating on peritoneal macrophages, *i*.*e*. CD11b^hi^ F4/80^hi^ cells (Fig. 2A, left panel), a shift in the fluorescence signal in co-cultures of PECs with pHrodo labeled RBCs occurs (Fig. 2A, right panel) indicating erythrophagocytosis by these cells. The shift in fluorescent signal expressed as delta median fluorescence intensity (ΔMFI) clearly shows that the peritoneal macrophages are the only cells involved in RBC uptake (Fig. 2B). Treatment of PECs with LPS enhances the erythrophagocytozing ability of these cells. Uptake of labeled RBCs by PECs was confirmed by fluorescence microscopy (Fig. 2C).

**Fig 3:**
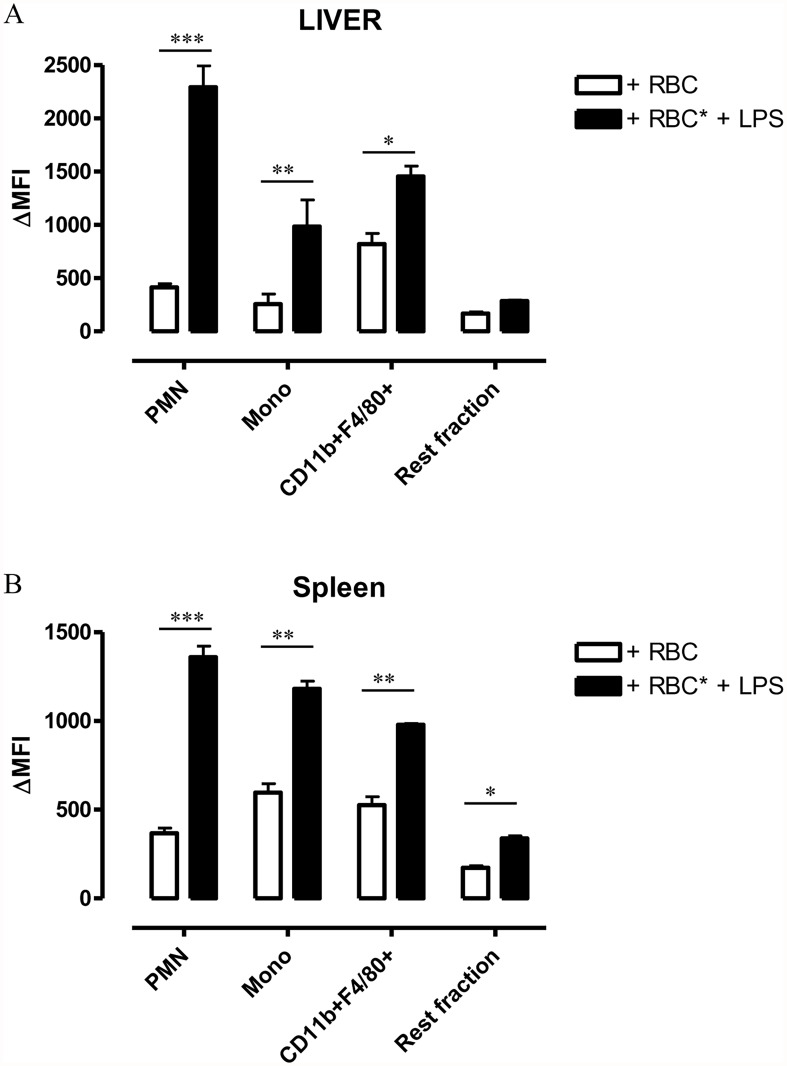
pHrodo erythrophagocytosis assay using naïve spleen and liver cells. Histograms showing delta medium fluorescence intensity (ΔMFI) obtained by subtracting the PE signal for cells incubated with unlabeled RBCs from cells incubated with pHrodo-labeled RBCs. Cells were either unstimulated (white bars) or stimulated with 1μg/ml LPS (black bars). Upper panel (A): Different liver cell populations (neutrophils, monocytes, CD11b^+^F4/80^+^ myeloid cells and Rest fraction) were identified as described in S1 Fig. Lower panel (B): Different spleen cell populations (neutrophils, monocytes, CD11b^+^F4/80^+^ myeloid cells and Rest fraction) were identified as described in S2 Fig. Results are presented +/- SEM and are representative of 3 independent experiments (for each point triplicates were used). Of note, *: p-values ≤ 0.05; **: p-values ≤ 0.01; ***: p-values ≤ 0.005 and if nothing is mentioned the differences were not significant.

Both liver and spleen are important organs for maintenance of RBC homeostasis and degradation of senescent RBCs under homeostatic conditions [23–25]. Hence, whole liver and spleen cultures were analyzed in the pHrodo erythrophagocytosis set-up to determine which cells are phagocytozing labeled RBCs. In the liver, CD11b^+^ F4/80^+^ myeloid cells (i.e. resident macrophages or Kupffer cells) were the most efficient erythrophagocytozing cells under steady state conditions (Fig. 3A, gating strategy described in S1 Fig A-D). In the spleen, CD11b^+^ F4/80^+^ myeloid cells (i.e. metallophilic and marginal zone macrophages) as well as CD11b^+^ Ly6C^+^ Ly6G^-^ monocytes and CD11b^+^ Ly6c^int^ Ly6G^+^ polymorphonuclear (PMN, granulocytes/neutrophils) cells were able to phagocytose RBCs under steady state conditions (Fig. 3B, gating strategy described in S2 Fig A-D). Of note, the Rest fraction of both spleen and liver, which consisted mainly of B- and T-cells and patrolling monocytes and NK cells respectively, did not exhibit significant erythrophagocytosis. In order to evaluate the process of erythrophagocytosis under inflammatory conditions, cells were stimulated with LPS *in vitro*. We observed that LPS stimulation of liver cells resulted in a 5.5-fold increase in RBC phagocytosis by the neutrophils, a 1.8 fold increase by the CD11b^+^ F4/80^+^ myeloid cells fraction and a 2.7 fold increase by the monocyte fraction (Fig. 3A). LPS stimulation of spleen cells resulted in a similar enhanced RBC phagocytosis by the neutrophils (3.7 fold increase), CD11b^+^ F4/80^+^ myeloid cells (1.8 fold increase) and monocytes (Fig. 3B) (3 fold increase).

### African trypanosomosis causes acute enhanced erythrophagocytosis in the liver and spleen

**Fig 4:**
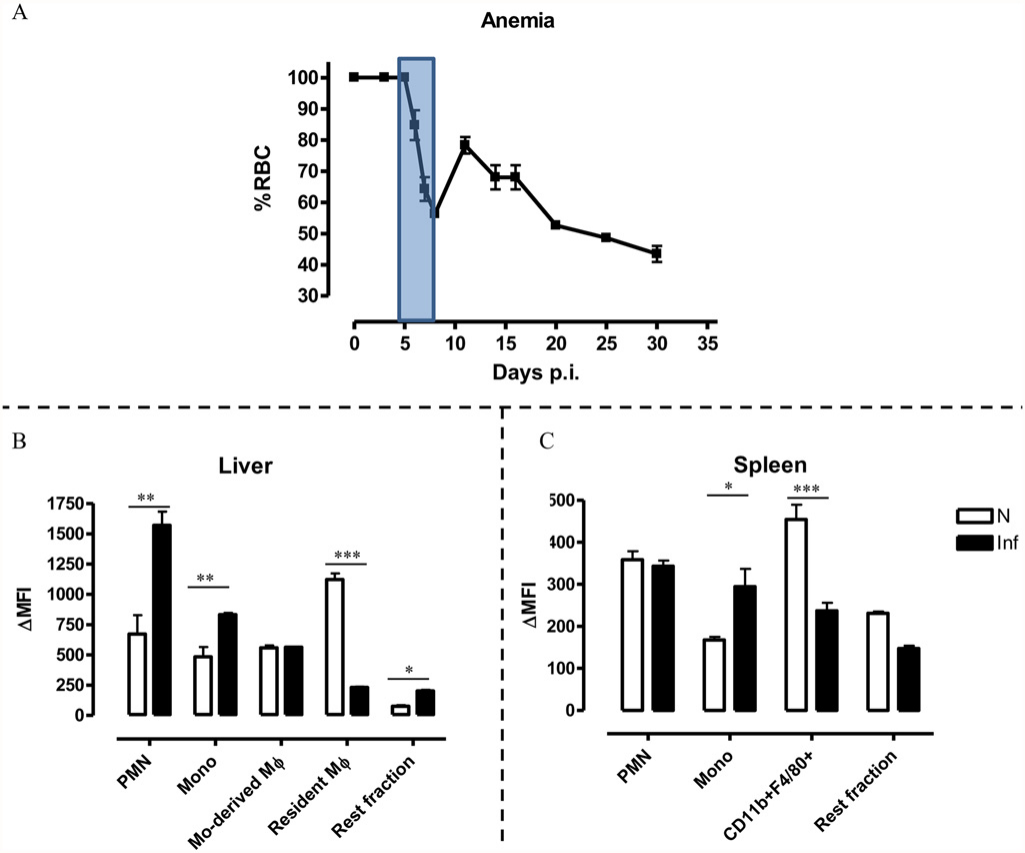
Anemia development during *T*. *brucei* infection and pHrodo erythrophagocytosis assay on liver and spleen cells during the acute stage of infection. Upper panel (A): Anemia profile during the course of *T*. *brucei* infection, whereby the boxed area indicates the time point of interest. Lower panels: Histograms showing delta medium fluorescence intensity (ΔMFI) obtained by subtracting the PE signal for cells in presence of unlabeled RBCs from cells in presence of pHrodo-labeled RBCs following *in vitro* incubation (B and C). Different cell populations (neutrophils, monocytes, CD11b^+^F4/80^+^ myeloid cells and Rest fraction) were identified for liver and spleen as described in S2 Fig and S3 Fig, respectively, from naïve (white bars) or *T*. *brucei* infected (day 6 p.i.) mice. Results are presented +/- SEM and are representative of 3 independent experiments (for each point triplicates were used). Of note, *: p-values ≤ 0.05; **: p-values ≤ 0.01; ***: p-values ≤ 0.005 and if nothing is mentioned the differences were not significant.

During the course of trypanosome infection anemia develops, which can be divided into different stages. During the acute stage of *T*. *b*. *brucei* infection, C57Bl/6 mice develop acute anemia, which is typically observed between day 5–8 post infection (Fig. 4A). After a slight recovery phase (i.e. between day 8–10), the reduction in the RBC percentage persists throughout the chronic phase of infection. Here, using the pHrodo *in vitro* erythrophagocytosis assay we determined whether enhanced erythrophagocytosis could be responsible for this severe reduction in RBCs observed during the early stage of infection. Whole liver and spleen cells of day 6 infected mice were put in co-culture with labeled RBCs of the corresponding mice and the erythrophagocytozing potential was analyzed and compared to that of non-infected animals. Trypanosome infection causes an alteration of the liver myeloid cell composition, therefore a more elaborate gating strategy (S3 Fig) allows to distinguish monocyte-derived macrophages (CD11b^+^ Ly6C^+^ MHC-II^+^) and resident macrophages (CD11b^+^ F4/80^+^ Ly6C^-^ MHC-II^+^) in addition to neutrophils and monocytes (CD11b^+^ Ly6C^+^ MHC-II^-^). In the liver, neutrophils and monocytes show a remarkable increase in their phagocytozing potential (Fig. 4B & S4 Fig) during infection while the phagocytozing potential of the monocyte-derived macrophages was unaltered. In contrast, the phagocytozing potential of resident macrophages diminished and the Rest fraction (consisting of NK cells and patrolling monocytes) only played a minor role in erythrophagocytosis during infection. In contrast to the situation in the liver, spleen neutrophil-mediated erythrophagocytosis was not enhanced upon infection (Fig. 4C). Monocytes on the other hand exhibited increased erythrophagocytosis and the CD11b^+^ F4/80^+^ myeloid cell-mediated erythrophagocytosis was reduced during infection (Fig. 4C).

When investigating the effect of *T*. *brucei* infection on erythrophagocytosis *in vitro* it appears that within the liver and spleen, neutrophils and monocytes are responsible for enhanced RBC clearance. The RBC clearance capacity of the liver Kupffer cells and spleen CD11b^+^ F4/80^+^ myeloid cells decreased at day 6 of infection.

**Fig 5:**
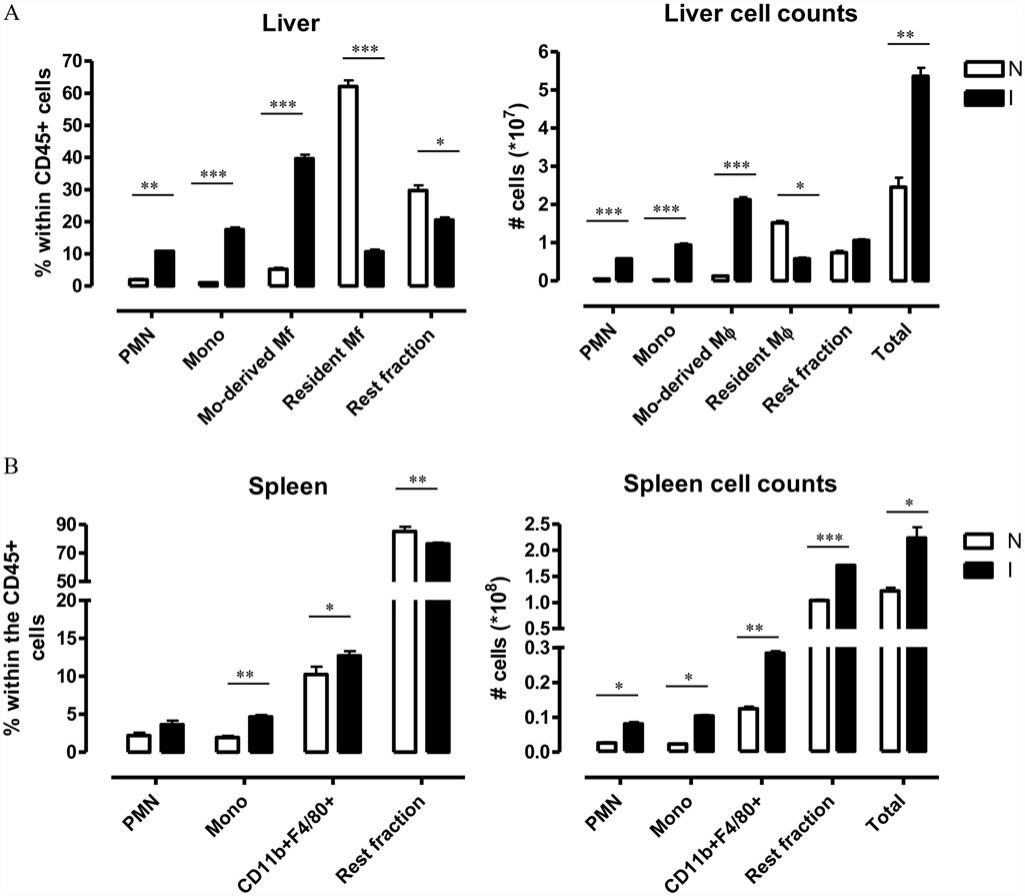
Different myeloid sub-populations expressed as percentage within the CD45+ hematopoietic compartment or as absolute numbers within the organ. Left panels: Percentage of the different cell populations (neutrophils/PMN, monocytes, CD11b^+^F4/80^+^ myeloid cells and Rest fraction) within the CD45+ hematopoietic compartment obtained following gating of liver (A) and spleen (B) as described in S1 Fig and S2 Fig, respectively, from non-infected (white bars) or *T*. *brucei* infected (day 6 p.i.) animals. Right panels: Absolute numbers of the different cell populations (neutrophils/PMN, monocytes, CD11b^+^F4/80^+^ myeloid cells and Rest fraction) in total liver and spleen of non-infected (white bars) and *T*. *brucei* infected (day 6 p.i.) mice. Results are presented +/- SEM and are representative of 3 independent experiments (for each point triplicates were used). Of note, *: p-values ≤ 0.05; **: p-values ≤ 0.01; ***: p-values ≤ 0.005 and if nothing is mentioned the differences were not significant.

Besides anemia development, trypanosomosis infection has been shown to have a vast effect on spleen and liver immune cell populations [26–28]. Indeed, as mentioned earlier we observed that during the early stage of infection there was an increase (in percentage as well as absolute numbers) of monocytic cells and neutrophils in the liver and a decrease in resident macrophages (Fig. 5A). In the spleen, a similar increase in monocytes and neutrophils is observed as well as an increase in CD11b^+^ F4/80^+^ myeloid cells (Fig. 5B). Therefore, at the level of the spleen it seems that besides monocytes also the CD11b^+^ F4/80^+^ myeloid cells (i.e. red pulp macrophages) can significantly contribute to erythrophagocytosis during infection. Hence both organs seem to contribute to erythrophagocytosis during the acute stage of infection.

### Red blood cells are altered during African Trypanosome infection

**Fig 6:**
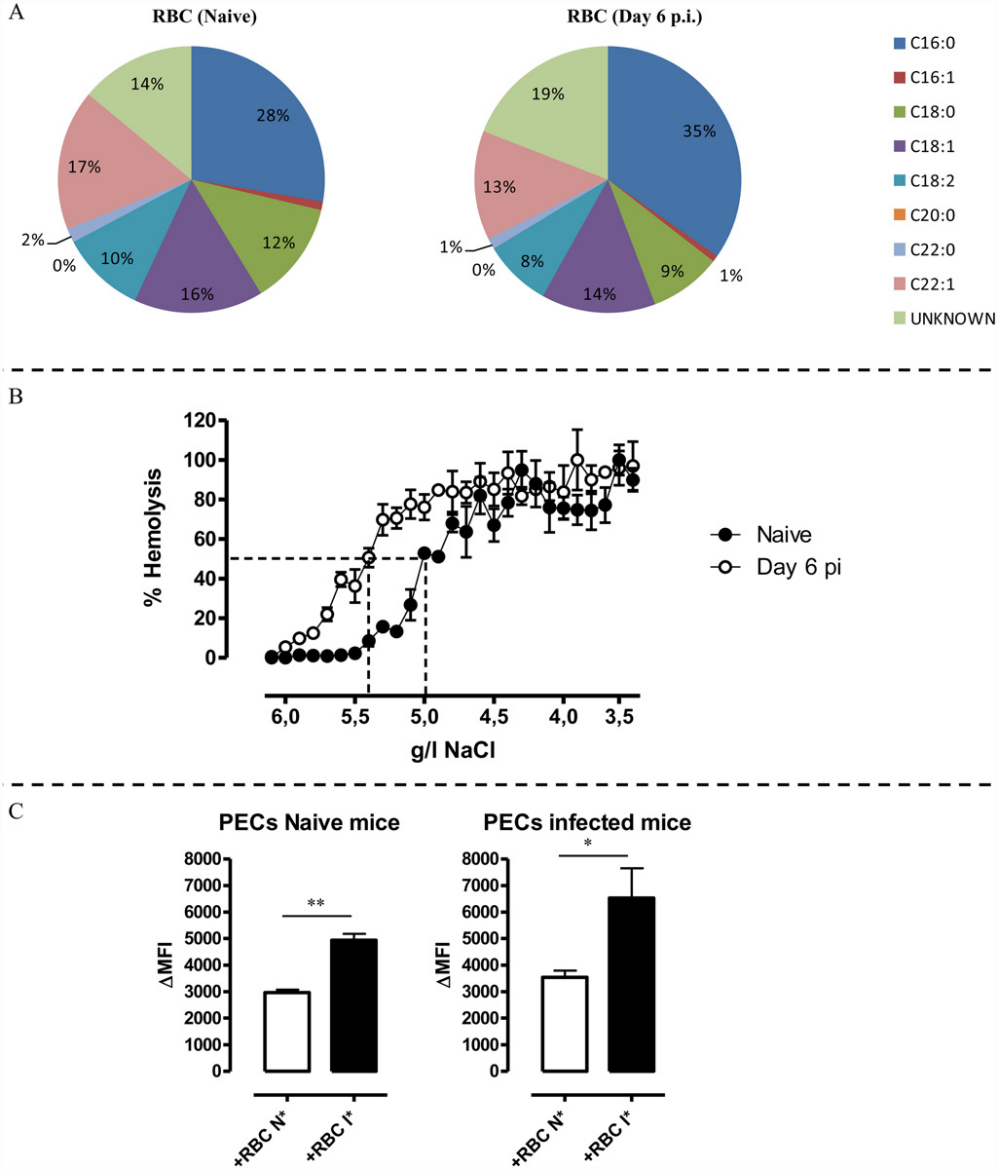
A. Chart representing the lipid composition of mature RBCs from naïve and *T*. *brucei* infected mice. This result is a representative of three independent experiments, including three mice per time point. B. Osmotic fragility profile of RBCs following incubation of naïve (black line) or *T*. *brucei* infected (day 6 p.i.) (white line) with decreasing concentrations of NaCl, resulting in hemolysis of RBCs. The percentage of hemolysis was plotted against the concentration of NaCl in the medium and the NaCl concentrations corresponding with 50% hemolysis were determined. As positive control RBCs were exposed to 100% distilled H_2_O and as negative control RBCs were exposed to 100% HBSS-solution. Results are representative of 3 independent experiments and expressed +/- SD. C. pHrodo erythrophagocytosis assay using RBCs from naïve and *T*. *brucei* infected mice co-cultured with PECs from non-infected (left panel) or infected (left panel) animals. Erythrophagocytosis by non-infected PECs of non-infected RBCs (white bars) or *T*. *brucei* infected (day 6 p.i.) (black bars). Results are presented +/- SEM and are representative of 3 independent experiments (for each point triplicates were used). Of note, *: p-values ≤ 0.05; **: p-values ≤ 0.01.

It is generally known that the amount of senescent RBCs increases during infection, resulting in an enhanced erythrophagocytosis. Hereby, the RBC membrane displays enormous plasticity and deformability to cope with changes in pressure and shear stress in the microcirculation [21]. 40% of the erythrocyte membrane is composed of lipids in the form of phospholipids, glycolipids and un-esterified cholesterol [23]. These lipids exchange easily with plasma lipoproteins by a continuous exchange mechanism [29]. Hence, serum cholesterol levels or, e.g. insertion of pathogen lipids during infection, influence the lipid composition of the erythrocyte membrane, altering its physical properties and hereby affecting RBC survival [21]. Therefore, an altered RBC membrane composition could also play a role in the induction of acute anemia during murine trypanosome infection. To assess this, a quantitative fatty acid determination was performed on FACS sorted RBCs from both non-infected and trypanosome infected animals (S5 Fig FACS gating). Fig. 6A shows that RBCs from infected animal (i.e. iRBC) have increased C16:0 (palmitate) fatty acids and reduced C18:0 (stearate), C18:1 (oleate), C18:2 (linoleate) and C22:1 (erucic acid) fatty acids compared to RBCs from non-infected animals, indicating that trypanosome infection indeed alters the RBC membrane composition. Next, the effect of trypanosomosis on RBC membrane rigidity was checked, using resistance to osmolarity changes as a readout. As indicated in Fig. 6B, a functional change in the RBC membranes of infected animals occurred, as the cells became significantly more susceptible to lysis. The assay was performed on day 6 post infection, concomitantly with the acute drop in RBC percentages. Of note, by comparing the RBC fragility of pHrodo labeled RBCs with unlabeled RBCs undergoing the same processed we could demonstrate that the pHrodo labeling had no effect on the RBC membrane rigidity neither for RBCs from non-infected or infected mice (S6 Fig).

Finally, the pHrodo *in vitro* erythrophagocytosis assay was used to assess a possible link between RBC membrane changes and susceptibility to accelerated erythrophagocytosis. Here, PECs from non-infected mice were co-cultured with pHrodo labeled RBCs from non-infected and trypanosome infected mice. PECs from non-infected mice showed an increased phagocytozing potential when co-cultured with RBCs from infected animals (ΔMFI: 4936 ± 235) compared to co-cultures with RBCs from non-infected animals (ΔMFI: 2970 ± 105), implying an altered RBC state during acute African Trypanosome infection (Fig. 6C, left panel).

In addition, it cannot be excluded that differences in the myeloid cell activation state occurring during infection can play a role in the enhanced RBC uptake. This suggestion was confirmed by the observation that PECs from infected mice showed an increased phagocytozing potential when co-cultured with RBCs from infected animals (ΔMFI: 6536 ± 1114) compared to co-cultures with RBCs from non-infected animals (ΔMFI: 3537 ± 264.1). In addition, the phagocytozing potential of PECs from infected mice was found to be higher than that of non-infected animals (Fig. 6C, right panel). Therefore, not only an altered RBC membrane state but also a difference in myeloid cell activation during acute African Trypanosome infection seems to contribute to enhance RBC uptake.

### The pHrodo *in vivo* erythrophagocytosis assay

**Fig 7:**
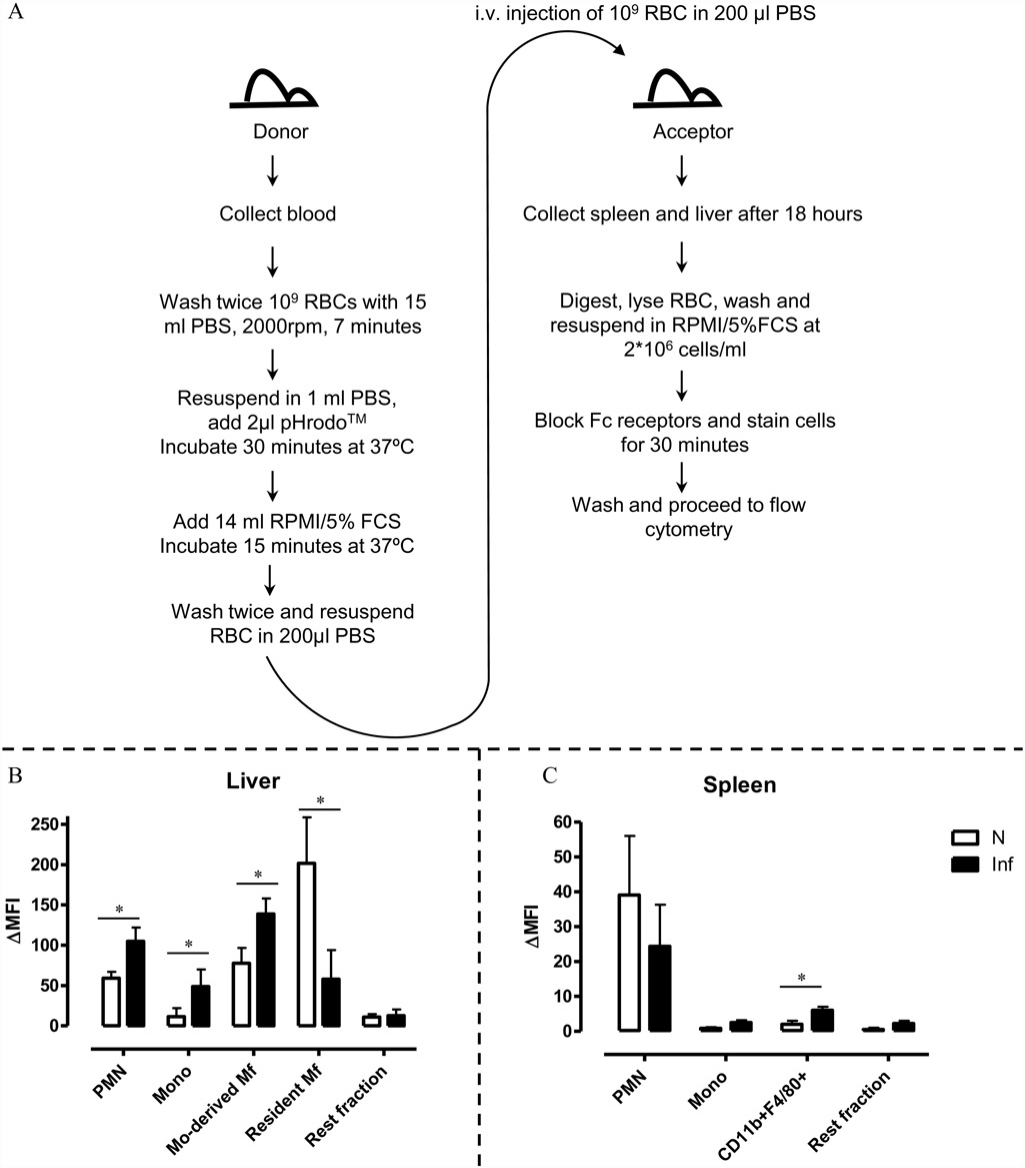
*In vivo* pHrodo erythrophagocytosis assay on liver and spleen cells during the acute stage of infection. A. Flow chart of the *in vivo* erythrophagocytosis assay protocol. To asses erythrophagocytosis in infected mice, donor mice should be sacrificed at day 5 post infection. Labeled blood is then transferred to 5 days-infected acceptor mice. At day 6 post infection the acceptor mice are sacrificed for organ isolation and flow cytometry analysis. As a control the same procedure is performed with naïve donor and acceptor mice. B. Erythrophagocytic potential of liver cell populations (neutrophils/PMN, monocytes, monocyte-derived macrophages, resident macrophages and rest fraction) from naïve (white bars) or *T*. *brucei* infected (day 6 p.i.). C. Erythrophagocytic potential of spleen cell populations (neutrophils/PMN, monocytes, CD11b^+^F4/80^+^ macrophages and rest fraction) from naïve (white bars) or *T*. *brucei* infected (day 6 p.i.) mice. Results are presented +/- SEM and are representative of 3 independent experiments (for each point triplicates were used). Of note, *: p-values ≤ 0.05 and if nothing is mentioned the differences were not significant.

In order to validate the biological significance of the *in vitro/ ex vivo* observations we setup an *in viv*o erythrophagocytosis assay (Fig. 7A), whereby pHrodo labeled RBCs from non-infected and infected mice were injected into non-infected or *T*. *brucei* infected (day 6 p.i.) mice respectively. Subsequently, 18 hours later the uptake of RBCs was tested by flow cytometry. To compare this new *in vivo* set up to a known technical approach, we performed a side-by-side experiment using RBC of Ubiquitin-GFP mice (i.e. GFP^+^RBC). When comparing these two systems we could establish that although both systems show Ter119+ signals when gating on liver resident macrophages of non-infected mice, only the pHrodo labeling system due to its pH-sensitivity allows to determine that indeed erythrophagocytosis is occurring (S7 Fig). As shown in Fig. 7B, in non-infected mice the resident macrophages were the main cells involved in RBC uptake. Yet, during the acute stage of infection, monocytes and monocyte-derived macrophages (collectively termed monocytic cells) as well as neutrophils exhibit enhanced erythrophagocytosis while the resident macrophages have a reduced erythrophagocytozing capacity. At the level of the spleen no significant enhanced RBC uptake was observed at the level of the monocytes and neutrophils, while there was a significant increase in erythrophagocytozing capacity of the CD11b^+^ F4/80^+^ myeloid cells (Fig. 7C). Collectively, these data indicate that liver monocytic cells and neutrophils as well as spleen CD11b^+^ F4/80^+^ myeloid cells exhibit an enhanced RBC uptake at this acute stage of infection and hence seem responsible for the induction of acute anemia.

## Discussion

Infection-associated anemia is considered one of the most important pathological features of trypanosomosis and constitutes a major cause of death in bovine African trypanosomosis [30]. Anemia is also a prominent pathological feature of murine trypanosome infection, offering a good model to identify the mechanisms that mediate this phenomenon. *T*. *b*. *brucei* infection of C57Bl/6 mice elicits a severe reduction of RBCs between day 5 and day 8 post infection. Given the acuteness of this phenomenon, a consumptive process seems to be implicated, in particular, as no hemolysis appears to occur. Previously, we have reported a strong increase of cell surface receptors involved in uptake of RBCs and iron-containing compounds in liver tissue. Therefore, liver-associated erythrophagocytosis mediated by cytokine-activated macrophages (M1 cells) is a likely contributor to the aggressive anemia during the acute phase of infection [12].

Multiple experimental set-ups have been used to study erythrophagocytosis, among which using sheep-opsonized RBCs [31] labeling of RBCs with 51Cr [32] or cell labeling with fluorescent cell tracking dyes such as PKH26 and carboxy-fluorescein succinimidyl ester (CFSE) [33–35]. With respect to the first assay, the disadvantages are the handling of radioisotopes and the fact that fresh sheep RBCs (foreign RBCs) need to be opsonized with IgG prior to incubation with phagocytes and phagocytozed RBCs need to be counted or measured in an ELISA reader. In addition, sheep RBCs need to be bought and the transport of the material might affect RBC-membrane stability [36]. Furthermore, this assay allows determining Fc-mediated RBC uptake and does not allow drawing any conclusions with respect to non-Fc mediated RBC uptake mediated via for instance CD36 (recognizing phosphatidylserine exposure on aged/damaged RBCs (SIRP1alpha/CD47) [37]. The downside to the use of the lipophilic membrane dye PKH26, which intercalates itself into the cell lipid bilayer, is that it could incorporate itself in other cells through a trogocytosis-like cell-cell membrane transfer mechanism, giving rise to specific binding and therefore masking ‘real’ phagocytosis [38,39]. Dyes such as CFSE which form random covalent bonds with amino groups on cellular proteins have an advantage regarding this drawback, but here it is impossible to distinguish real endocytosed RBCs from RBCs sticking to the outside of the macrophage membrane. Using instead RBCs labeled with the cell permeable dye Calcein AM [40] or RBCs from Green fluorescent protein (GFP)+/+ mice entail the same disadvantage regarding the distinguishing of surface bound or phagocytozed RBCs.

In each of the above-mentioned RBC uptake setups there is an overestimation of the real erythrophagocytosis and therefore the RBC-specific Ter119 antibody needs to be included to allow distinguishing between surface-bound or phagocytozed RBCs. Other phagocytosis assays using labeled dextran beads, do not represent the real *in vivo* situation and only reveal the phagocytozing potential without giving specific information about erythrophagocytosis. In the past, we designed an *in vitro* adherent liver cell co-culture assay whereby a monolayer of liver cells was plated *in vitro* and incubated with a monolayer of RBCs. Clearance plaques were then visualized and counted and served as an indication of erythrophagocytosis [13]. The downside of this assay is that it is impossible to discriminate between erythrophagocytosis and RBC lysis. We now used a different setup to study erythrophagocytosis whereby we labeled RBCs with pHrodo, an amine-reactive ester dye that forms covalent bonds with proteins on the RBCs. This dye has been used successfully before for the labeling of phagocytozed apoptotic lymphocytes [41] and platelets [42]. pHrodo labeling offers the advantage that it is only fluorescent at acidic conditions, hence it will be fluorescent in the phagolysosome (pH 4.5–5) but not in the cytoplasm (pH 7–7.4) or on the outside of the cell. We confirmed, using a comparative study of GFP^+^RBCs from ubiquitin-GFP mice and pHrodo-labeled RBCs, that the latter technique allows a more specific determination of erythrophagocytosis (see S7 Fig). In addition, we showed that the pHrodo labeling procedure did not affect the RBC membrane rigidity of either non-infected or infected animals. Using a co-culturing system of pHrodo labeled RBCs from non-infected mice with PECs, spleen or liver cells we could observe via flow cytometry and immunofluorescence microscopy that indeed RBCs are taken up by myeloid cells. Furthermore, the phagocytozing potential can be increased following LPS-stimulation. To validate the assay in a biological relevant situation the pHrodo labeled RBCs were *ex vivo* put in co-culture with whole spleen or liver cell suspensions from non-infected or infected (day 6 p.i.) animals. We observed that in the liver resident macrophages or Kupffer cells seem to be the most prominent erythrophagocytozing cells during steady state situations. However, during the acute phase of infection the erythrophagocytozing capacity of these cells decreased. Yet, an alternative explanation might rely in the fact that these myeloid cells have already taken up a lot of RBCs *in vivo* and hence cannot take up much more RBCs *in vitro*. In addition, also in absolute numbers the amount of Kupffer cells decreased, which might be due to massive apoptosis [43]. In contrast, the erythrophagocytozing capacity of monocytes and neutrophils was significantly enhanced during infection. Moreover, given that at this stage of infection there is a massive influx of monocytic cells and neutrophils in the liver, these cells can significantly contribute to anemia development. Of note, the influx of monocytic cells into the liver during the early stages of infection has been reported before and was shown to contribute to parasite control via release of pro-inflammatory molecules such as TNF on one hand and pathogenicity development (liver damage) on the other [27,44]. It has also been shown that the myeloperoxidase activity (MPO) increased drastically during the acute stage of infection [45] which infers that indeed neutrophils become more activated already at the early stages of infection. In the spleen, all myeloid cells were able to phagocytose RBCs during steady state situations. Yet, during infection the erythrophagocytosis ability seems to be unaltered for splenic neutrophils, decreased for CD11b^+^ F4/80^+^ myeloid cells (i.e. red pulp macrophages) and increased for monocytes. However, when taking into account the total number of cells, both organs can significantly contribute to phagocytosis of RBCs.

In a second validation set-up, an *in vivo* pHrodo based erythrophagocytosis assay was established and shown to corroborate largely the *in vitro/ex vivo* obtained results. Yet, the phagocytic capacity of liver monocyte-derived macrophages as well as splenic CD11b^+^ F4/80^+^ myeloid cells was underestimated in the *ex vivo* approach. A possible explanation for this discrepancy could be that in the *ex vivo* assay conditions (*i*.*e*. 18 hours of incubation) the cells from infected animals are more susceptible to apoptosis, while in the *in vivo* setup cells are measured directly after isolation.

Taken together, it seems that activation of both liver and spleen neutrophils and monocytic cells, as well as splenic CD11b^+^F4/80^+^ myeloid cells leads to enhanced erythrophagocytosis and hence can explain the occurrence of severe acute-stage non-hemolytic anemia observed in *T*. *brucei* trypanosomosis.

Besides differences in erythrophagocytozing potential occurring during infection at the level of the myeloid cells, we also observed that RBCs from infected animals are phagocytozed more efficiently than non-infected RBCs, suggesting an alteration of the RBC membrane during trypanosome infection. Upon analysis of the RBCs, we observed an enhanced osmolytic fragility and an altered fatty acid membrane composition in RBCs from day 6 infected mice compared to non-infected WT mice. Hence, it could be that the modification of RBC properties contributes to enhanced RBC uptake by phagocytozing cells. Interestingly, in the *T*. *evansi* model it has been shown that lipid peroxidation causes membrane injury and osmotic fragility resulting in RBC destruction [46]. In *T*. *congolense* infection on the other hand, anemia has been correlated to RBC de-galactosylation [47]. In the *T*. *vivax* model on the other hand it has been shown that parasite-derived trans-sialidases released during the acute phase of infection triggers erythrophagocytosis by desialylating the major surface erythrocytes sialoglycoproteins, the glycophorins [40]. Besides differences in RBC membrane composition, we also observed that the activation state of myeloid cells plays also an important role in the observed enhanced RBC uptake. Hence, both RBC membrane deformability and enhanced myeloid cells activation can contribute to enhanced RBC uptake.

In conclusion, the pHrodo labeling method for RBCs used herein provides a sensitive, reproducible and accurate method for the determination of both *ex vivo* and *in vivo* erythrophagocytosis. Results obtained indicate that infiltrating monocytic cells and neutrophils are undergoing infection-associated hyper-activation, resulting in increased erythrophagocytosis of membrane-modified RBCs. This coincides with the appearance of a type 1 cytokine storm that has been described to hallmark the first wave of trypanosomosis control. Given that (i) the percentage of resident macrophages within the CD45 hematopoietic compartment of the liver decreases drastically and coincides with reduced erythrophagocytozing potential, that (ii) there is a massive influx of monocytic cells and neutrophils into the liver and spleen that exhibit an enhanced erythrophagocytozing activity, and (iii) that splenic CD11b^+^F4/80^+^ myeloid cells exhibit enhanced erythrophagocytosis we propose that liver and spleen-associated monocytic cells and neutrophils as well as splenic CD11b^+^ F4/80^+^ myeloid cell activation plays a previously underestimated role in the acute phase of anemia development.

## Supporting Information

S1 Fig Representative liver gating strategy. Selection of (A) CD45^+^ cells based on a FSC-A/CD45 profile following gating on single cells (SSC-A/FSC-W profile) within the life gate (FSC-A/SSC-A profile); (B) CD11b versus Ly6c profile within the CD45^+^ population allows detection of CD11b^+^Ly6c^+^ myeloid cells. (C) Ly6c versus Ly6G profile within the CD11b^+^Ly6c^+^ population allows the identification of CD11b^+^Ly6c^int^Ly6G^+^ (neutrophils/PMN) and CD11b^+^Ly6c^high^Ly6G^-^ (monocytes) cells. (D) CD11b versus F4/80 profile within the remaining (omitting the CD11b^+^Ly6c^+^ population) population from profile B allows the identification of CD11b^+^F4/80^+^ myeloid cells.
(TIF)

S2 Fig Representative spleen gating strategy. Selection of (A) CD45^+^ cells based on a FSC-A/CD45 profile following gating on single cells (SSC-A/FSC-W profile) within the life gate (FSC-A/SSC-A profile); (B) CD11b versus Ly6c profile within the CD45^+^ population allows detection of CD11b^+^Ly6c^+^ myeloid cells. (C) Ly6c versus Ly6G profile within the CD11b^+^Ly6c^+^ population allows the identification of CD11b^+^Ly6c^int^Ly6G^+^ (neutrophils/PMN) and CD11b^+^Ly6c^high^Ly6G^-^ (monocytes) cells. (D) CD11b versus F4/80 profile within the remaining (omitting the CD11b^+^Ly6c^+^ population) population from profile B allows the identification of CD11b^+^F4/80^+^ myeloid cells.
(TIF)

S3 Fig Representative liver gating strategy used for the *in vivo* erythrophagocytosis assay as well as surface marker expression for non-infected (A) and *T*. *brucei* infected (B) mice. First, CD45^hi^ cells were selected based on a FSC-A/CD45 profile followed by gating on single cells (SSC-A/FSC-W profile) within the life gate (FSC-A/SSC-A profile); (B) CD11b versus CD11b^+^ cells using an CD11b/FSC-A profile within the CD45^+^ population. Subsequently, neutrophils (CD11b^+^Ly6c^int^Ly6G^+^/PMN) were identified using a Ly6G/FSC-A profile and the remaining cells were used in an Ly6C versus MHC-II profile to identify monocytes (CD11b^+^Ly6c^high^Ly6G^-^MHC-II^-^), monocyte-derived macrophages (CD11b^+^Ly6c^high^Ly6G^-^MHC-II^+^), resident macrophages (CD11b^+^Ly6c^-^Ly6G^-^MHC-II^+^) and a Rest fraction (CD11b^+^Ly6c^-^Ly6G^-^MHC-II^-^). Surface markers used on these identified population were F4/80, CCR2, Ly6B, MerTK and CD64.
(TIF)

S4 Fig Representative histograms showing the pHrodo signal on different liver cell populations following injection of pHrodo-labeled or unlabeled RBCs. Using the same gating strategy as in S3 Fig, PMN, monocytes, monocyte-derived macrophages, resident macrophages and a Rest fraction were identified for the non-infected (upper panels) and infected (lower panels) mice. Mϕ: macrophage. PE/phycoerythrine represents the pHrodo signal.
(TIF)

S5 Fig Typical FACS profile on blood from non-infected or *T*. *brucei* infected (day 6 p.i.) mice following gating on Ter119 positive RBCs. The profile of CD71 versus Ter119 allows identifying immature (Ter119^+^ CD71^+^) and mature (Ter119^+^ CD71^-^) RBCs. Only mature RBCs were sorted and used for RBC lipid analysis (Fig. 5A).
(TIF)

S6 Fig Osmotic fragility profile of RBCs used in the labeling procedure. LP: Labeling procedure. Profile of pHrodo-labeled (white dots) or unlabeled (black dots) RBCs following incubation of non-infected (left panel) or *T*. *brucei* infected (day 6 p.i., right panel) with decreasing concentrations of NaCl, resulting in hemolysis of RBCs. The percentage of hemolysis was plotted against the concentration of NaCl in the medium and the NaCl concentrations corresponding with 50% hemolysis were determined. As positive control, RBCs were exposed to 100% distilled H_2_O and as negative control RBCs were exposed to 100% HBSS-solution. Results are representative of 2 independent experiments and expressed +/- SD.
(TIF)

S7 Fig Representative FACS profile from non-infected mice following injection of labeled RBCs and gating on liver resident macrophages. The same gating strategy as in S3 Fig was used, whereby we selected liver resident macrophages exhibiting the highest phagocytosing capacity in steady-state. Upper section: FITC (GFP, upper) and PE (pHrodo, lower) signals of naïve mice injected with GFP+RBCs (left panels) or pHrodo-labeled RBCs (right panels). Lower section: Histograms showing delta medium fluorescence intensity (ΔMFI) obtained by subtracting the FITC or PE signal for cells in presence of unlabeled RBCs from cells in presence of GFP+ or pHrodo-labeled RBCs, respectively, following *in vivo* injection. The Ter119 signal is obtained by subtracting the signal of mice receiving only PBS from mice receiving RBCs. Results are presented +/- SEM and are representative of 2 independent experiments (for each point triplicates were used). Of note, *: p-values ≤ 0.05 and **: p-values ≤ 0.01.
(TIF)
