# Supplementary figures and images for: Development of a pHrodo-Based Assay for the Assessment of In Vitro and In Vivo Erythrophagocytosis during Experimental Trypanosomosis
Source: PLoS Negl Trop Dis. 2015 Mar 5;9(3):e0003561. doi: 10.1371/journal.pntd.0003561 (PMC4352936; doi:10.1371/journal.pntd.0003561)

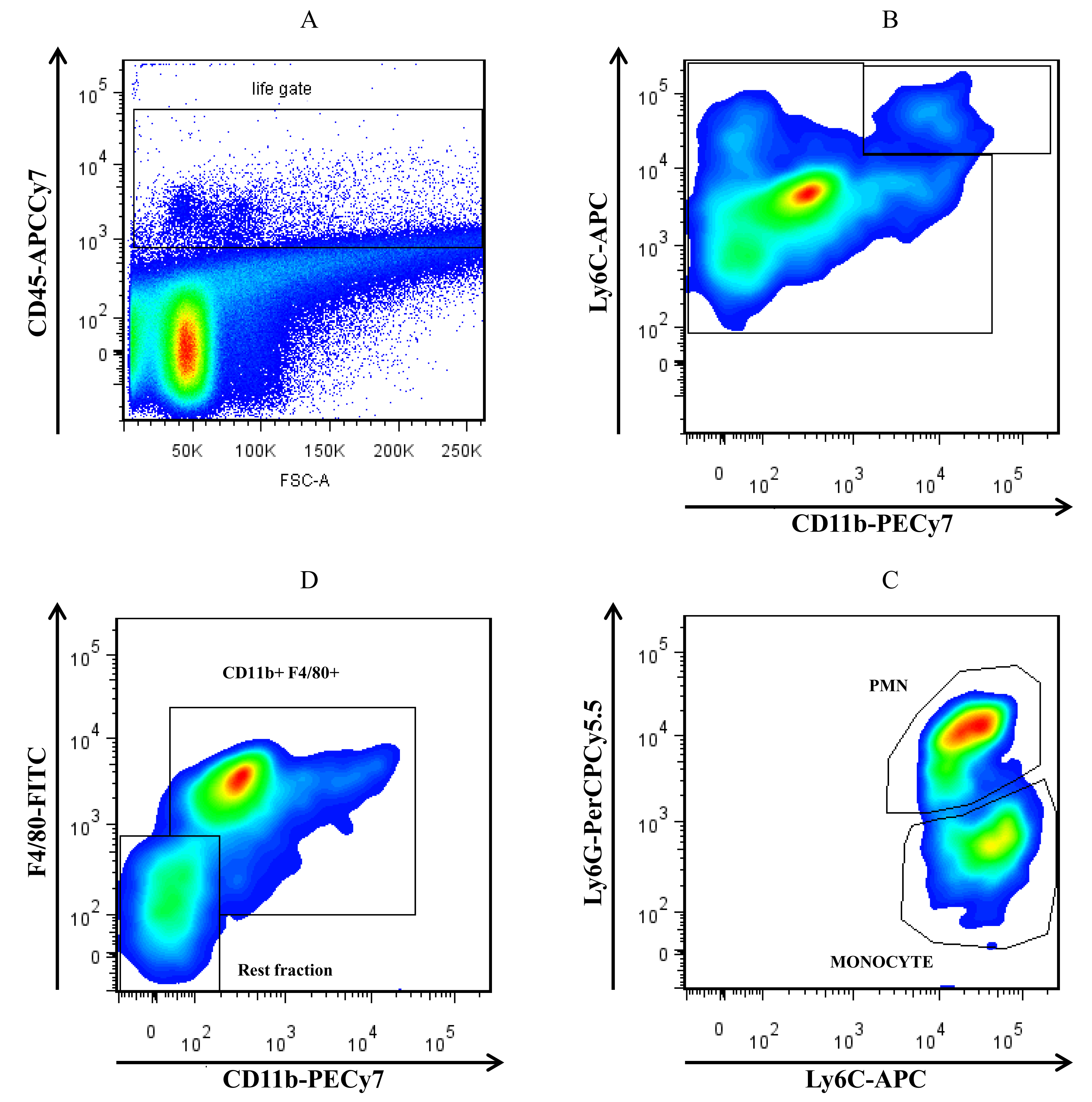

Supplement: S1 Fig — Selection of (A) CD45+ cells based on a FSC-A/CD45 profile following gating on single cells (SSC-A/FSC-W profile) within the life gate (FSC-A/SSC-A profile); (B) CD11b versus Ly6c profile within the CD45+ population allows detection of CD11b+Ly6c+ myeloid cells. (C) Ly6c versus Ly6G profile within the CD11b+Ly6c+ population allows the identification of CD11b+Ly6cintLy6G+ (neutrophils/PMN) and CD11b+Ly6chighLy6G- (monocytes) cells. (D) CD11b versus F4/80 profile within the remaining (omitting the CD11b+Ly6c+ population) population from profile B allows the identification of CD11b+F4/80+ myeloid cells. (TIF) [file pntd.0003561.s001.tif]

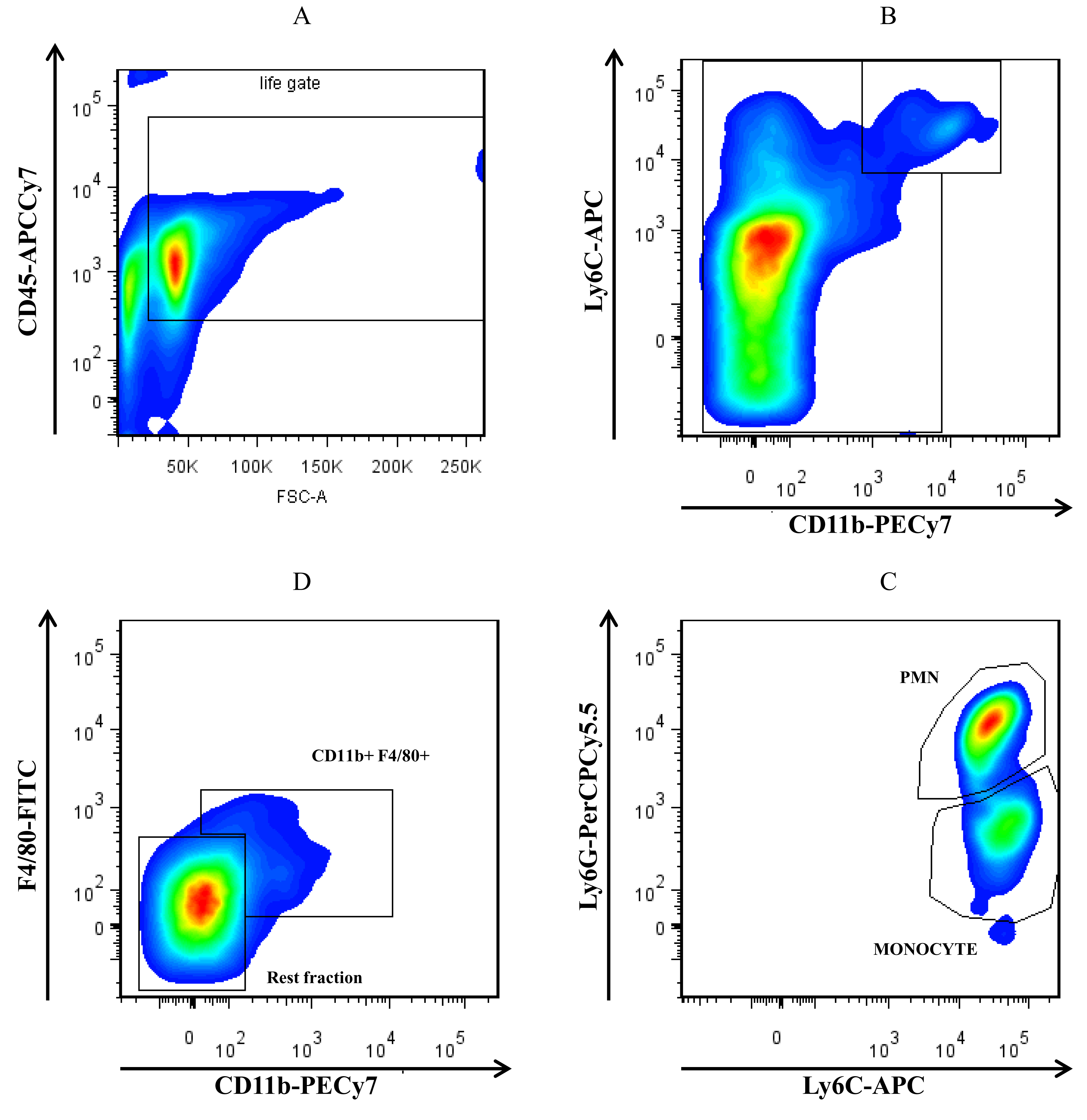

Supplement: S2 Fig — Selection of (A) CD45+ cells based on a FSC-A/CD45 profile following gating on single cells (SSC-A/FSC-W profile) within the life gate (FSC-A/SSC-A profile); (B) CD11b versus Ly6c profile within the CD45+ population allows detection of CD11b+Ly6c+ myeloid cells. (C) Ly6c versus Ly6G profile within the CD11b+Ly6c+ population allows the identification of CD11b+Ly6cintLy6G+ (neutrophils/PMN) and CD11b+Ly6chighLy6G- (monocytes) cells. (D) CD11b versus F4/80 profile within the remaining (omitting the CD11b+Ly6c+ population) population from profile B allows the identification of CD11b+F4/80+ myeloid cells. (TIF) [file pntd.0003561.s002.tif]

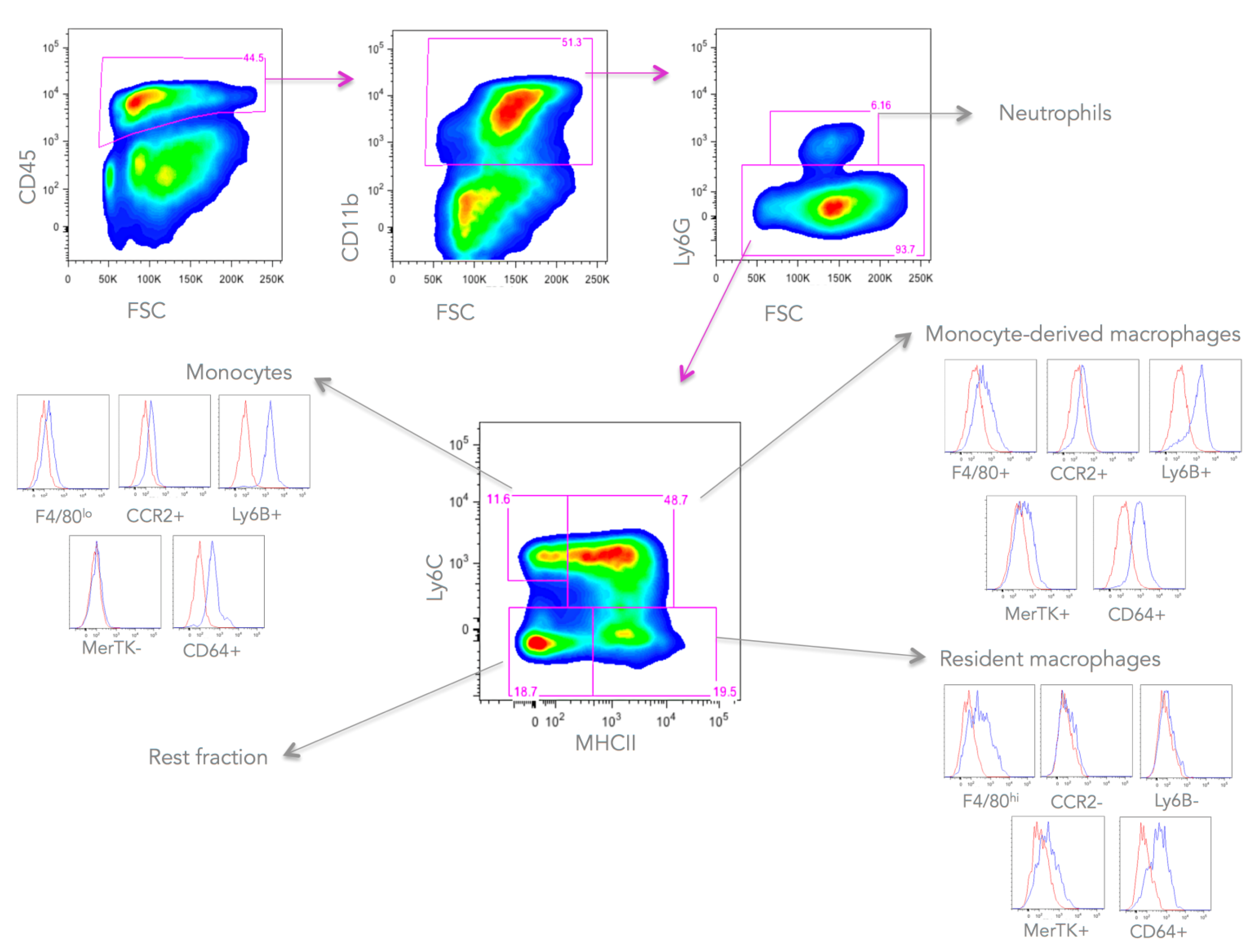

Supplement: S3 Fig — First, CD45hi cells were selected based on a FSC-A/CD45 profile followed by gating on single cells (SSC-A/FSC-W profile) within the life gate (FSC-A/SSC-A profile); (B) CD11b versus CD11b+ cells using an CD11b/FSC-A profile within the CD45+ population. Subsequently, neutrophils (CD11b+Ly6cintLy6G+/PMN) were identified using a Ly6G/FSC-A profile and the remaining cells were used in an Ly6C versus MHC-II profile to identify monocytes (CD11b+Ly6chighLy6G-MHC-II-), monocyte-derived macrophages (CD11b+Ly6chighLy6G-MHC-II+), resident macrophages (CD11b+Ly6c-Ly6G-MHC-II+) and a Rest fraction (CD11b+Ly6c-Ly6G-MHC-II-). Surface markers used on these identified population were F4/80, CCR2, Ly6B, MerTK and CD64. (TIF) [file pntd.0003561.s003.tif]

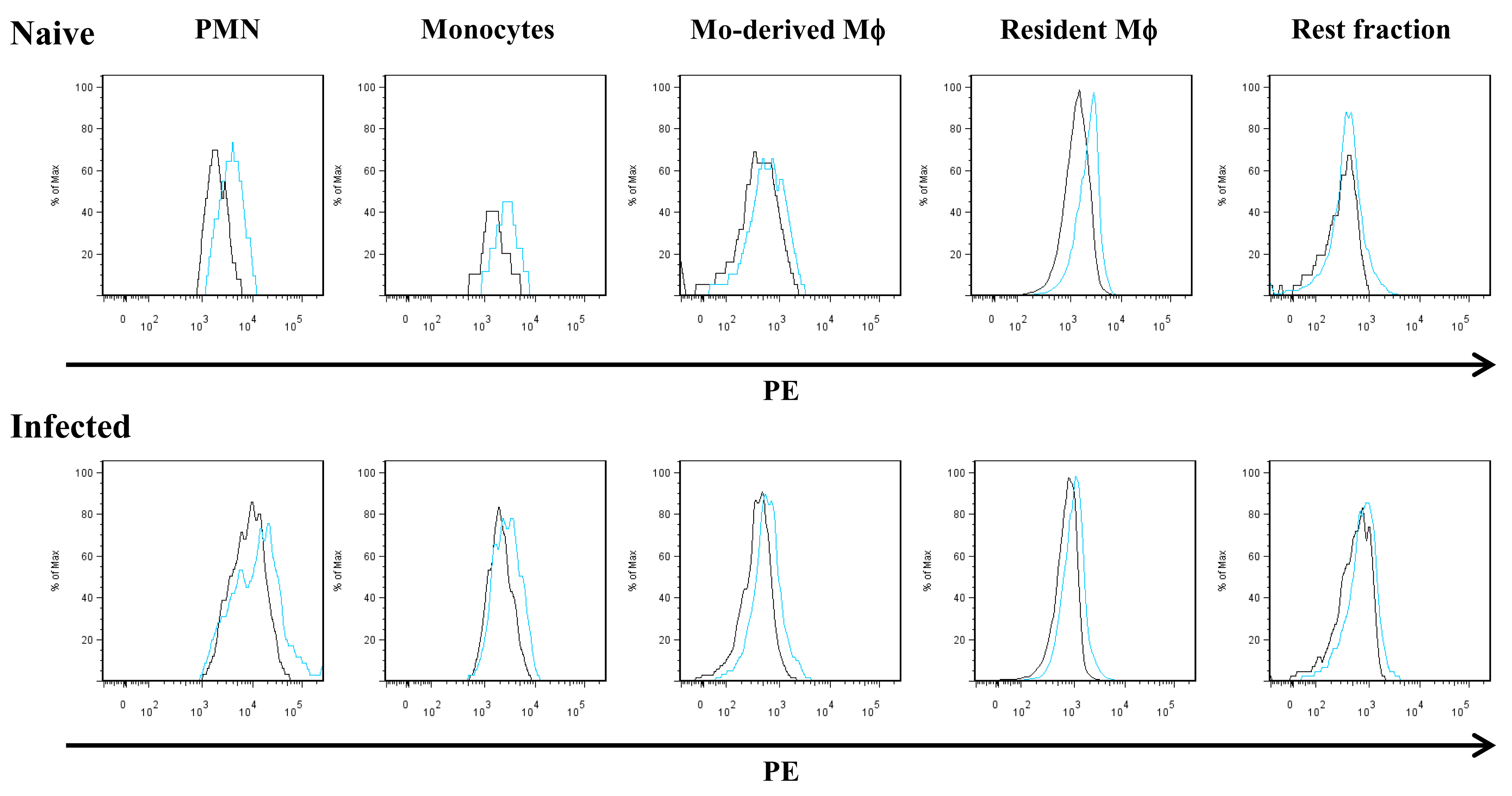

Supplement: S4 Fig — Using the same gating strategy as in S3 Fig, PMN, monocytes, monocyte-derived macrophages, resident macrophages and a Rest fraction were identified for the non-infected (upper panels) and infected (lower panels) mice. Mϕ: macrophage. PE/phycoerythrine represents the pHrodo signal. (TIF) [file pntd.0003561.s004.tif]

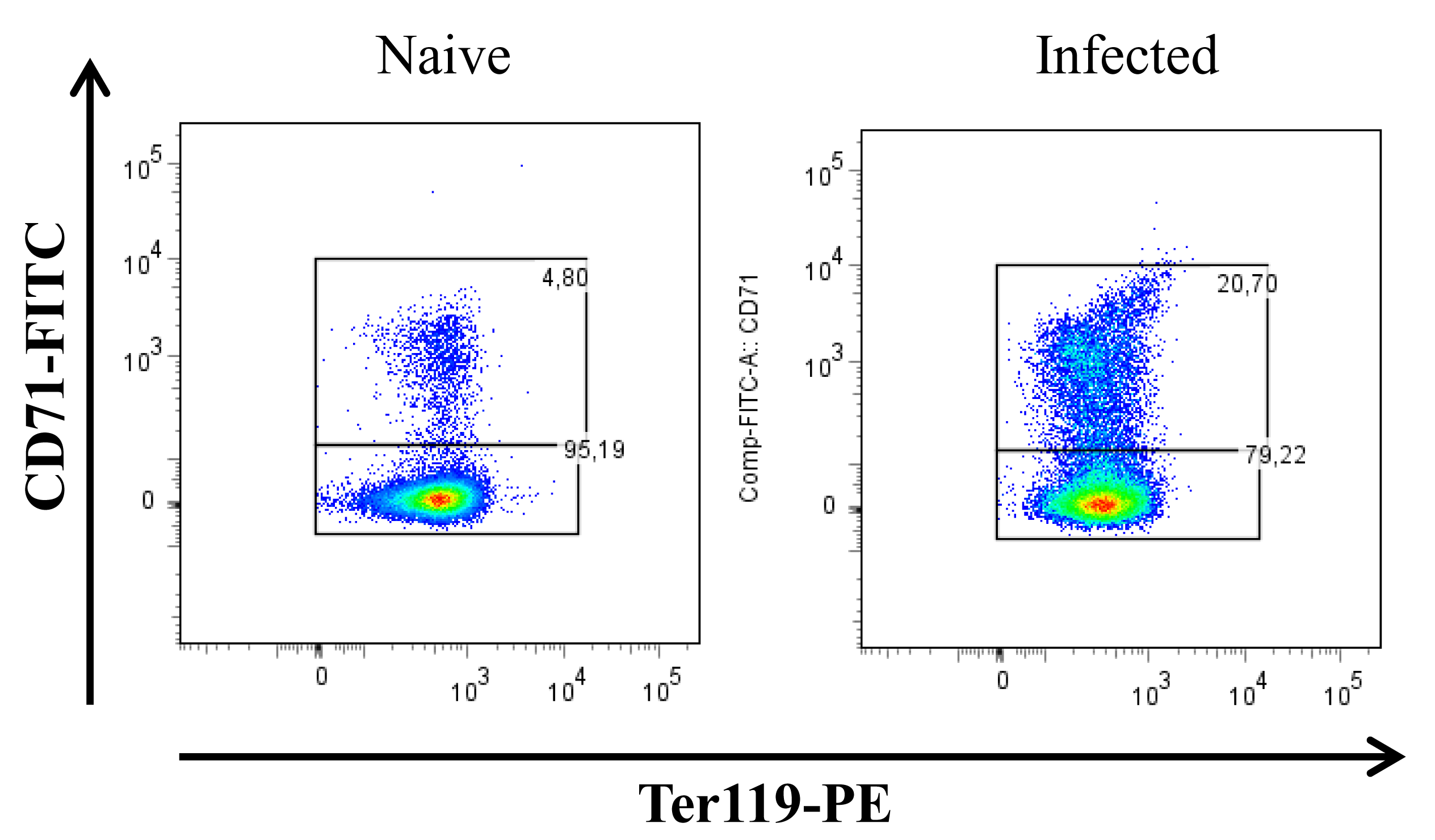

Supplement: S5 Fig — The profile of CD71 versus Ter119 allows identifying immature (Ter119+ CD71+) and mature (Ter119+ CD71-) RBCs. Only mature RBCs were sorted and used for RBC lipid analysis (Fig. 5A). (TIF) [file pntd.0003561.s005.tif]

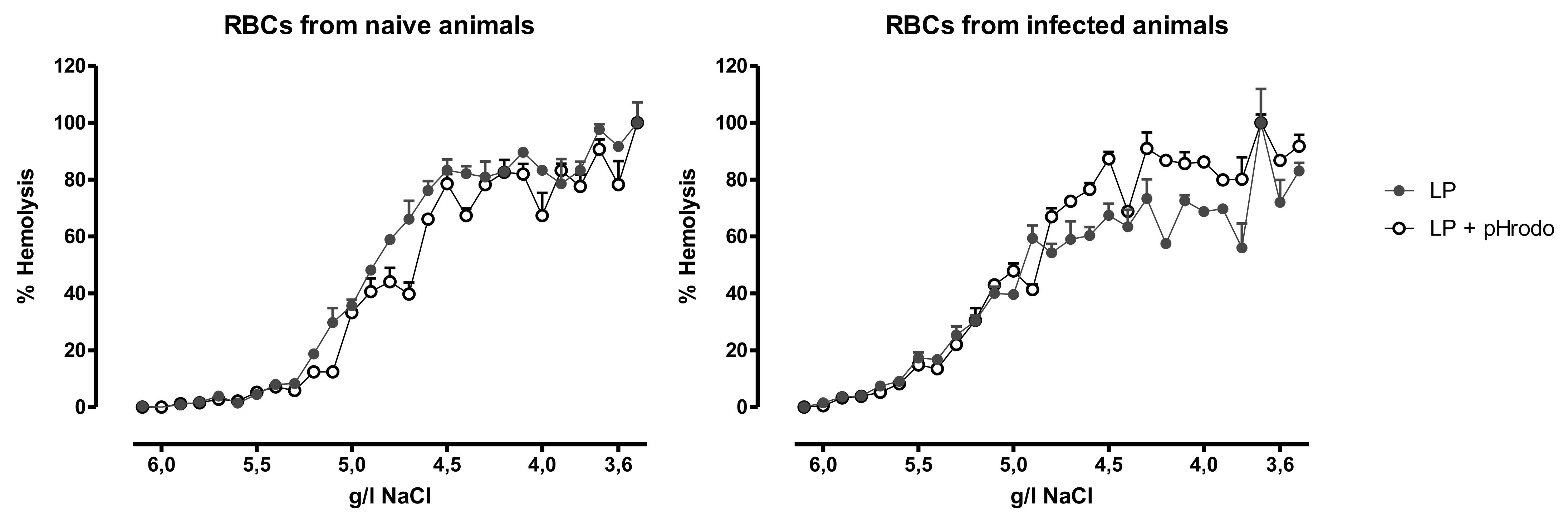

Supplement: S6 Fig — LP: Labeling procedure. Profile of pHrodo-labeled (white dots) or unlabeled (black dots) RBCs following incubation of non-infected (left panel) or T. brucei infected (day 6 p.i., right panel) with decreasing concentrations of NaCl, resulting in hemolysis of RBCs. The percentage of hemolysis was plotted against the concentration of NaCl in the medium and the NaCl concentrations corresponding with 50% hemolysis were determined. As positive control, RBCs were exposed to 100% distilled H2O and as negative control RBCs were exposed to 100% HBSS-solution. Results are representative of 2 independent experiments and expressed +/- SD. (TIF) [file pntd.0003561.s006.tif]

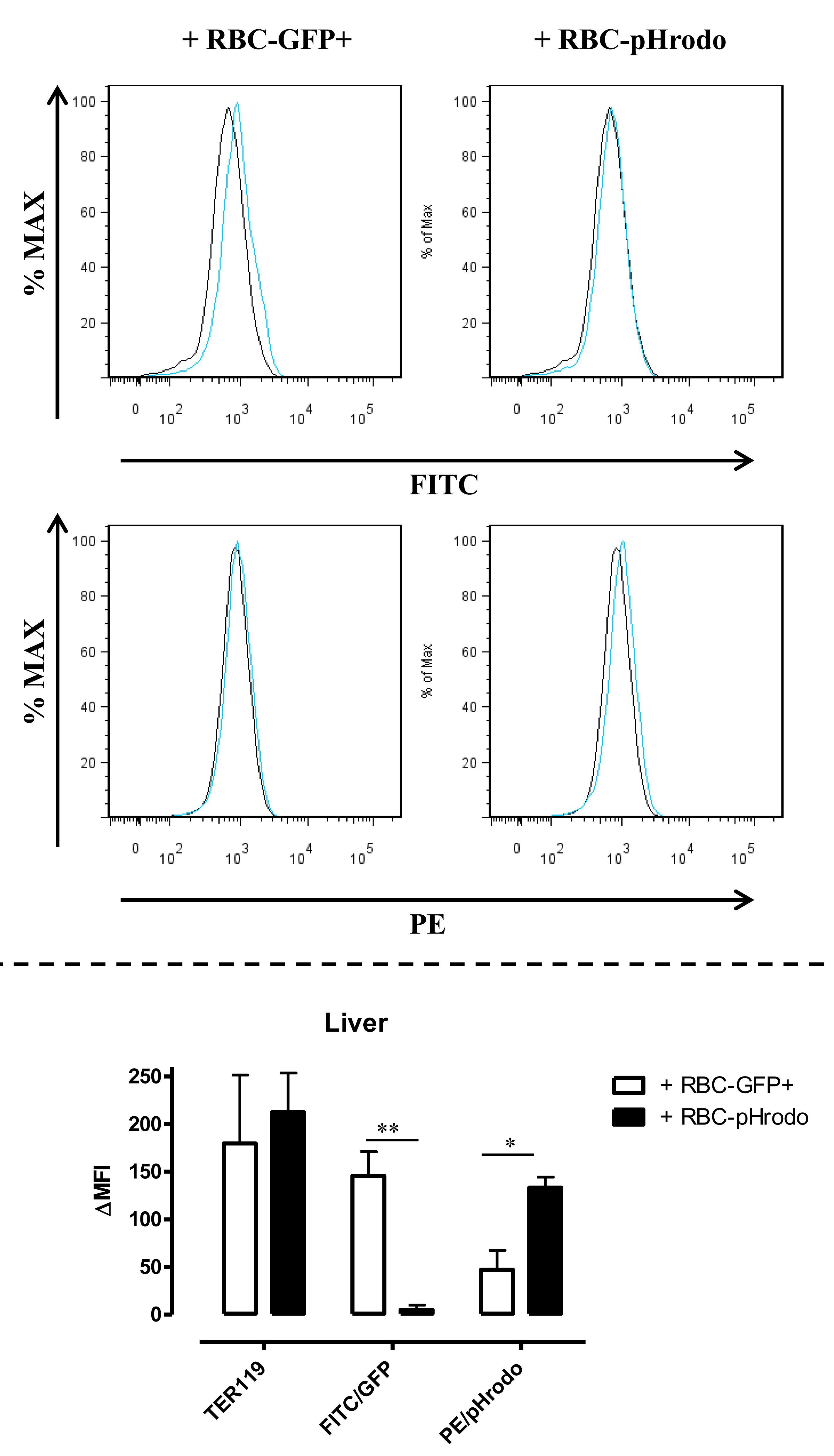

Supplement: S7 Fig — The same gating strategy as in S3 Fig was used, whereby we selected liver resident macrophages exhibiting the highest phagocytosing capacity in steady-state. Upper section: FITC (GFP, upper) and PE (pHrodo, lower) signals of naïve mice injected with GFP+RBCs (left panels) or pHrodo-labeled RBCs (right panels). Lower section: Histograms showing delta medium fluorescence intensity (ΔMFI) obtained by subtracting the FITC or PE signal for cells in presence of unlabeled RBCs from cells in presence of GFP+ or pHrodo-labeled RBCs, respectively, following in vivo injection. The Ter119 signal is obtained by subtracting the signal of mice receiving only PBS from mice receiving RBCs. Results are presented +/- SEM and are representative of 2 independent experiments (for each point triplicates were used). Of note, *: p-values ≤ 0.05 and **: p-values ≤ 0.01. (TIF) [file pntd.0003561.s007.tif]
